# Lipid metabolism in type 1 and type 2 diabetes: A comparative cross-sectional study

**DOI:** 10.1097/MD.0000000000046758

**Published:** 2025-12-26

**Authors:** Fatemah Saleh Bin Saleh, Ahmed R. Alibrahim, Awad S. Alshahrani, Aida Aldughaither, Moeber M. Mahzari, Weam S. Alharbi, Ghadah B. Alanazi

**Affiliations:** a Department of Family Medicine, King Abdulaziz Medical City, Ministry of National Guard Health Affairs, Riyadh, Saudi Arabia; b Division of Endocrinology and Metabolism, King Abdulaziz Medical City, Riyadh, Saudi Arabia; c College of Medicine, King Saud Bin Abdulaziz University for Health Sciences, Riyadh, Saudi Arabia; d Department of Family Medicine, Ministry of Health, Riyadh, Saudi Arabia.

**Keywords:** cardiovascular risk, diabetes mellitus, dyslipidemia, glycemic control, lipid abnormalities

## Abstract

Types 1 and 2 diabetes mellitus (T1DM and T2DM, respectively) are chronic conditions with distinct pathophysiological mechanisms and clinical manifestations. Dyslipidemia is a common complication in both types, contributing to increased cardiovascular risk. This study aimed to compare the demographic and laboratory characteristics of patients with T1DM and T2DM in Saudi Arabia. We conducted 2 different studies both are retrospective cross-sectional studies that involved 409 T2DM and 509 T1DM patients, respectively. Data were collected from electronic medical records. Clinical characteristics, including diabetes duration, body mass index, hemoglobin A1C levels, and lipid profiles, were analyzed. Statistical comparisons were conducted to identify significant differences between the 2 groups. Patients with T2DM were older (mean age, 58.93 years) than those with T1DM (mean age, 26.15 years). Additionally, substantial demographic differences were observed between the 2 groups. Compared with patients with T2DM, who had hemoglobin A1C levels between 7% to 9%, patients with T1DM had worse glycemic control, with 55.8% of them having such values. Patients with T2DM were more likely to have obesity (63.6%) than those with T1DM (21.8%). Compared with patients with T2DM, those with T1DM had lower triglyceride levels but higher mean low-density lipoprotein, high-density lipoprotein, and total cholesterol levels. A considerably larger proportion of individuals with T2DM had aberrant triglyceride and low high-density lipoprotein levels, indicating a higher incidence of dyslipidemia. Patients with T1DM and T2DM have different clinical characteristics and lipid abnormalities. Patients with T2DM are at higher risk of dyslipidemia and related cardiovascular complications. To reduce cardiovascular risk in patients with diabetes, customized therapies that concentrate on lipid management and glycemic control are necessary. To manage dyslipidemia, further studies are necessary to investigate the underlying mechanisms and develop efficient treatment plans.

## 1. Introduction

Diabetes mellitus is a chronic metabolic disease characterized by abnormalities in insulin action, secretion, or both, which lead to persistent hyperglycemia.^[1,2]^ Types 1 and 2 diabetes mellitus (T1DM and T2DM, respectively) are the 2 primary forms of the disease.^[3,4]^ Although the pathophysiological causes and clinical manifestations of each type differ, all can result in serious complications if left untreated.^[4]^ Dyslipidemia, a prevalent disorder in patients with diabetes, considerably increases the cardiovascular risk associated with the disease. Therefore, monitoring and managing lipid profiles is a crucial aspect of diabetes management.^[5,6]^

In T1DM, the immune system targets and destroys the pancreatic beta cells that produce insulin.^[7]^ This results in a complete lack of insulin.^[7]^ T1DM can occur at any age; however, it usually manifests in childhood or adolescence. To manage blood glucose levels, patients with T1DM need lifelong insulin therapy.^[7]^ In patients with T1DM-associated dyslipidemia, low-density lipoprotein cholesterol (LDL-C), triglycerides, and total cholesterol levels are often elevated, while high-density lipoprotein cholesterol (HDL-C) levels are typically decreased.^[8,9]^ These anomalies are worsened by poor glycemic management, which increases the risk of cardiovascular disease (CVD).^[9]^

The main risk factors for T2DM are insulin resistance, a condition in which the body’s cells lose their sensitivity to insulin, and a relative insulin deficit.^[10]^ T2DM is closely associated with poor dietary habits, obesity, physical inactivity, and hereditary factors.^[10]^ Although T2DM usually manifests in adulthood, it is becoming more common in younger populations.^[11]^ In contrast to T1DM, T2DM is frequently managed with dietary changes, oral hypoglycemic medications, and, occasionally, insulin therapy.^[12]^ Compared with T1DM, dyslipidemia in T2DM is usually more severe and manifests as an increase in small, dense LDL particles, decreased HDL-C levels, and increased triglyceride levels.^[13]^ Patients with T2DM have an increased risk of CVD, largely attributable to this lipid profile, referred to as atherogenic dyslipidemia.^[13]^

Although dyslipidemia is linked to both forms of diabetes, the patterns and underlying mechanisms differ. The main causes of lipid abnormalities in T1DM are inadequate insulin production and poor glycemic control.^[8]^ On the other hand, central obesity, hypertension, and insulin resistance are major contributors to metabolic syndrome and T2DM-associated dyslipidemia.^[13]^ With T2DM, the lipid profile is usually more atherogenic, which raises the risk of cardiovascular events. Understanding the distinctions in lipid profiles between T1DM and T2DM is essential for formulating customized treatment plans aimed at reducing the risk of CVD. A crucial component of comprehensive diabetes care involves managing dyslipidemia with medication, lifestyle changes, and optimal glycemic control. Further research into the mechanisms underlying these lipid abnormalities and their impact on cardiovascular outcomes will improve our capacity to properly monitor and treat diabetic dyslipidemia.

Despite the extensive literature on dyslipidemia in diabetes, there is limited research specifically characterizing lipid profile variations between T1DM and T2DM in Saudi Arabia. Given the unique genetic, lifestyle and environmental factors affecting the Saudi population, understanding these differences is essential for preventive and therapeutic strategies. This study aims to bridge this gap by analyzing and comparing lipid profile patterns among Saudi patients with T1DM and T2DM, providing data that can enhance region-specific clinical management of dyslipidemia and cardiovascular risk in diabetic individuals.

## 2. Materials and methods

The included data was extracted from 2 previous published studies conducted by the authors.^[14,15]^ Both studies were retrospective cross-sectional studies conducted to ensure a thorough comparison of lipid profiles in patients with T1DM and T2DM. The study was conducted in accordance with ethical guidelines, ethical approval was obtained from the Institutional Review Board of King Abdullah International Medical Research Center Approval #NRRC21R/054/02 and #RC20/583/R. Additionally, patient data were handled with strict confidentiality, and all necessary ethical considerations were followed.

The study was conducted in the Ministry of National Guard Health Affairs outpatient clinics in 3 primary healthcare centers and King Abdulaziz Medical City clinics in Riyadh, Saudi Arabia. Three primary health care were included: National Guard Comprehensive Specialized Center, King Abdul-Aziz City Housing (Iskan Yarmouk) Center, and Health Care Specialty Center. These facilities serve a large population by offering general outpatient care, including family medicine clinics for the follow-up of chronic diseases.

Simple random selection was used to select the sample from a list of patients who satisfied the data warehouse’s inclusion/exclusion criteria (BESTCare 2.0); for the first study, patients were selected using a random integer generator available online. Data from the Department of Family Medicine indicated that 240,000 people visited the 3 sites, of whom approximately two-thirds were adults (160,000) with T2DM. According to estimates, 10% of the population had T2DM as per the 2019 annual report of the Family Medicine and Primary Health Care Department, and 31% of patients with T2DM also had dyslipidemia. The estimated sample size using OpenEpi (https://www.openepi.com) was calculated with a 95% confidence level, yielding 409 patients for the study.

In the second study, a retrospective chart review was conducted at King Abdulaziz Medical City in Riyadh, a part of the Ministry of National Guard Health Affairs, for the T1DM group that met the inclusion criteria.

The inclusion criteria of T2DM patients were as follows: adults aged over 35 years who were diagnosed with T2DM, visited primary care facilities in 2019 and 2020, had medical records with more than 75% of the data accessible, and had lipid profile and hemoglobin A1C (HbA1c) data available. Patients who were pregnant, had a family history of high cholesterol or receiving medications such as thiazide diuretics, beta-blockers, lipid-lowering agents, or hormonal therapy were excluded.

The inclusion criteria of T1DM patients were as follows: adults aged 18 to 64 years with a T1DM diagnosis who were followed up at King Abdulaziz Medical City clinics in Riyadh between January 1, 2016, and December 31, 2020. Patients without a lipid profile; those with other types of diabetes mellitus, hypothyroidism, nephrotic syndrome, chronic liver disease, or a history of smoking or alcohol consumption; non-Saudis; and those receiving medications such as thiazide diuretics, beta-blockers, lipid-lowering agents, or hormonal therapy were excluded.

Similar variables were extracted for both groups which include demographics like age and gender, in addition to laboratory testing like HbA1c and fasting lipid profile all were obtained from medical records. In the absence of pancreatic autoantibody testing, the diagnosis of T1DM was established clinically based on the patient’s abrupt onset of symptoms, presentation of diabetic ketoacidosis, and noticeably increased HbA1c levels.

For both groups, lipid abnormalities were determined using the National Cholesterol Education Program (III) criteria. Abnormalities were determined as follows for the T2DM group: triglycerides > 200 mg/dL, total cholesterol > 240 mg/dL, LDL > 160 mg/dL, and HDL < 40 mg/dL.^[16,17]^ Triglycerides > 1.7 mmol/L, HDL < 1.55 mmol/L, LDL > 2.59 mmol/L, and total cholesterol > 5.18 mmol/L were considered lipid abnormalities for the T1DM group.^[15]^ Dyslipidemia was diagnosed based on an abnormal lipid value. The 3 categories for HbA1c were poor (>10%), suboptimal (7–10%), and ideal (<7%).

A spreadsheet was used to record the data, and IBM SPSS Statistics for Windows, Version 24.0 (IBM Corp., Armonk), was used for analysis. Categorical variables are presented as frequencies and percentages, while numerical variables are presented as mean ± standard deviation. Chi-square tests were used to compare categorical variables, while independent samples *t*-tests were used to examine numerical data between groups. Statistical significance was set at *P* < .05.

To account for potential confounders influencing lipid levels independently of diabetes type, additional analyses were conducted.

## 3. Results

Data were collected for 509 and 409 patients with T1DM and T2DM, respectively. Table 1 presents the clinical characteristics for the patients with T1DM and T2DM. Overall, equal proportions of males and females were observed across the T1DM and T2DM groups. However, a significantly higher proportion of older patients (>40 years) was observed among those with T2DM (97.1%) than among those with T1DM (5.5%) (*P* < .001). Regarding the duration of diabetes, approximately 60% of the patients with T1DM had been diagnosed for >10 years, compared with 51.8% of those with T2DM (*P* < .04). More than half (55.8%) of the patients with T1DM had HbA1c values between 7% and 9% compared with 43.0% of those with T2DM (*P* < .001). A significantly higher proportion of patients with T2DM had obesity (63.6%) than those with T1DM (21.8%) (*P* < .001).

**Table 1:** Demographic characteristics of the participants with T1DM and T2DM.

Parameters	Total n (%)	T1DM n (%) (N = 509)	T2DM n (%) (N = 409)	*P*-value
Sex				
Male	335 (36.5)	192 (37.7)	143 (35.0)	.38
Female	583 (63.5)	317 (62.3)	266 (65.0)	
Age				
<40 years	493 (53.7)	481 (94.5)	12 (2.9)	<.001
≥40 years	425 (46.3)	28 (5.5)	397 (97.1)	
Duration of diabetes				
<5 years	105 (11.4)	51 (10.0)	54 (13.2)	.04
5–10 years	295 (32.1)	152 (29.9)	143 (35.0)	
>10 years	518 (56.4)	306 (60.1)	212 (51.8)	
HbA1c				
<7%	146 (15.9)	56 (11.0)	90 (22.0)	<.001
7–9%	460 (50.1)	284 (55.8)	176 (43.0)	
>9%	312 (33.9)	169 (33.2)	142 (34.7)	
Weight category (BMI)				
Underweight	41 (4.5)	40 (7.9)	1 (0.2)	<.001
Normal weight	244 (26.6)	217 (42.6)	27 (6.6)	
Overweight	262 (28.5)	141 (27.7)	121 (29.6)	
Obese	371 (40.4)	111 (21.8)	260 (63.6)	

BMI = body mass index, HbA1c = hemoglobin A1C, T1DM = type 1 diabetes mellitus, T2DM = type 2 diabetes mellitus.

Weight categories: underweight = <18.5 kg/m^2^, normal weight = 18.5–24.9 kg/m^2^, overweight = 25.0–29.9 kg/m^2^, obese = ≥30.0 kg/m^2^.

Table 2 presents the demographic and clinical characteristics of the patients with T1DM and T2DM. The mean ages were 26.15 and 58.93 years for patients with T1DM and T2DM, respectively. While males with T1DM were slightly younger than females with T1DM (*P* = .02), no significant difference in age was observed between males and females with T2DM (*P* = .41). The mean body mass index (BMI) for males with T1DM was significantly lower than that for females with T1DM (*P* < .001), and a similar pattern was observed between males and females with T2DM (*P* < .001). However, no significant differences in the mean HbA1c were observed between males and females with T1DM and T2DM (Table 2). While the mean LDL was nearly the same for males and females with T1DM (*P* = .76), the mean LDL was significantly higher for females with T2DM than for males (*P* = .01). The mean HDL was significantly higher in females with T1DM and T2DM than in males (*P* < .001). However, the mean triglyceride levels (mmol/L) were significantly higher in males with T1DM and T2DM than in females (Table 2).

**Table 2:** Demographic and clinical characteristics of patients with T1DM and T2DM (presented as mean ± SD).

	T1DM				T2DM			
Parameters	Total (n = 509)	Male (n = 192)	Female (n = 317)	*P*-value	Total (n = 409)	Male (n = 143)	Female (n = 266)	*P*-value
Age (yr)	26.15 ± 71.2	25.21 ± 7.15	26.72 ± 7.05	.02	58.93 ± 10.61	58.34 ± 11.22	59.24 ± 10.28	.41
BMI (kg/m^2^)	25.74 ± 5.65	24.44 ± 5.03	26.52 ± 5.86	<.001	32.50 ± 6.30	30.01 ± 4.75	33.83 ± 6.62	<.001
HbA1c (%)	8.92 ± 4.64	9.26 ± 7.25	8.70 ± 1.67	.19	8.53 ± 1.82	8.44 ± 1.64	8.58 ± 1.90	.43
LDL (mmol/L)	3.03 ± 0.83	3.05 ± 0.87	3.02 ± 0.81	.76	2.91 ± 0.96	2.73 ± 0.88	3.01 ± 0.99	.01
HDL (mmol/L)	1.38 ± 0.32	1.26 ± 0.27	1.46 ± 0.32	<.001	1.14 ± 0.41	1.02 ± 0.21	1.20 ± 0.47	<.001
Total chol (mmol/L)	4.68 ± 0.86	4.60 ± 0.95	4.74 ± 0.80	.08	4.52 ± 1.07	4.34 ± 1.01	4.61 ± 1.09	.01
Triglycerides (mmol/L)	0.91 ± 0.52	0.97 ± 0.61	0.87 ± 0.45	.03	1.76 ± 0.96	1.99 ± 1.27	1.64 ± 0.72	<.001

BMI = body mass index, HbA1c = hemoglobin A1C, HDL = high-density lipoprotein, LDL = low-density lipoprotein, T1DM = type 1 diabetes mellitus, T2DM = type 2 diabetes mellitus, total chol = total cholesterol.

Table 3 shows the prevalence of lipid abnormalities by sex in the T1DM and T2DM groups. An approximately equal proportion of males and females with T1DM had abnormal LDL (*P* = .87) and total cholesterol values (*P* = .12). However, a significantly higher proportion of females with T2DM (62.8%) had abnormal LDL values than males with T2DM (51.0%) (*P* = .02). Overall, a significantly higher proportion of males with T1DM and T2DM had abnormally low HDL values than females (*P*-value < .001). However, no significant differences in total cholesterol values were observed between male and female patients with T1DM and T2DM.

**Table 3:** Prevalence of lipid abnormalities, stratified by sex, in patients with T1DM (categorical).

	T1DM				T2DM			
Lipid type	Total n (%)	Male n (%)	Female n (%)	*P*-value	Total n (%)	Male n (%)	Female n (%)	*P*-value
LDL								
Normal	156 (30.6)	58 (30.2)	98 (30.9)	.87	169 (41.3)	70 (49.0)	99 (37.2)	.02
Abnormal	353 (69.4)	134 (69.8)	219 (69.1)		240 (58.7)	73 (51.0)	167 (62.8)	
HDL								
Normal	467 (91.7)	163 (84.9)	304 (95.7)	<.001	20 (4.9)	2 (1.4)	18 (6.8)	.01
Abnormal	42 (8.3)	29 (15.1)	42 (8.3)		389 (95.1)	141 (98.6)	248 (93.2)	
Total chol								
Normal	392 (77.0)	155 (80.7)	237 (74.8)	.12	310 (75.8)	114 (79.7)	196 (73.7)	.17
Abnormal	117 (23.0)	37 (19.3)	117 (23.0)		99 (24.2)	29 (20.3)	70 (26.3)	
Triglycerides								
Normal	484 (94.9)	178 (92.7)	306 (96.5)	.05	233 (57.0)	72 (50.3)	161 (60.5)	.04
Abnormal	25 (4.9)	14 (7.3)	11 (3.5)		176 (43.0)	71 (49.7)	105 (39.5)	

HDL = high-density lipoprotein, LDL = low-density lipoprotein, total chol = total cholesterol.

Tables 4a and 4b demonstrate the prevalence of lipid abnormalities among patients with T1DM and T2DM, stratified by age. For patients with T1DM, no significant differences in lipid parameters were found between the 2 age groups (all *P* > .05). However, a greater proportion of patients with T2DM aged <40 years had abnormal LDL values (91.7) than older patients with T2DM (57.7%) (*P* = .02). Similarly, a significantly higher proportion of patients with T2DM aged <40 years had abnormal total cholesterol values than older patients with T2DM (22.4%) (*P* < .001). However, similar to patients with T1DM, no significant differences in HDL and triglycerides were observed between the 2 age groups.

Table 5 presents a comparison of lipid profiles (mean values) between patients with T1DM and those with T2DM. Overall, the mean LDL (*P* = .03), HDL (*P* < .001), and total cholesterol (*P* = .01) levels were significantly higher in patients with T1DM than in those with T2DM. However, the mean triglyceride levels were lower in patients with T1DM than in those with T2DM (*P* < .001) (Table 5).

**Table 5:** Comparison of lipid profiles (numerical) between patients with T1DM and T2DM.

Lipid profile	T1DM (n = 509)	T2DM (n = 409)	*P*-value
LDL (mmol/L)	3.03 ± 0.83	2.90 ± 0.96	.03
HDL (mmol/L)	1.38 ± 0.32	1.14 ± 0.41	<.001
Total chol (mmol/L)	4.68 ± 0.86	4.52 ± 1.07	.01
Triglycerides (mmol/L)	0.91 ± 0.51	1.76 ± 0.96	<.001

HDL = high-density lipoprotein, LDL = low-density lipoprotein, T1DM = type 1 diabetes mellitus, T2DM = type 2 diabetes mellitus, total chol = total cholesterol.

## 4. Discussion

This study examined the lipid profiles and clinical characteristics of patients with T1DM and T2DM in specialized and primary healthcare facilities in Saudi Arabia. Our research adds to the existing body of knowledge on diabetes management and complications by shedding light on demographic disparities, prevalence of dyslipidemia, and variations in lipid parameters between patients with T1DM and T2DM.

The demographic and clinical features of individuals with T1DM and T2DM differed significantly. The T2DM group had a considerably larger percentage of older patients (>40 years) than the T1DM group (5.5%), indicating a notable difference in the age distribution. This is in line with the natural course of these conditions, wherein T2DM is more common in older adults and T1DM usually first manifests in infancy or adolescence.^[18,19]^ Differences in the duration of diabetes were also observed. A greater proportion of patients with T1DM (60%) had a disease duration of more than 10 years than patients with T2DM (51.8%). This discrepancy may be attributed to the earlier onset of T1DM.^[20,21]^

HbA1c values, a measure of glucose management, revealed notable variations between the 2 groups. Over half (55.8%) of patients with T1DM had HbA1c values in the range of 7% to 9%, compared with 43% of patients with T2DM. Furthermore, compared with patients with T1DM (11%), a greater percentage of patients with T2DM (22%) had HbA1c levels below 7%, indicating improved glycemic control. This result is consistent with earlier research showing that the complete lack of endogenous insulin production in T1DM makes achieving optimal glycemic control more challenging.^[22–24]^

Significant variations in BMI and obesity rates between the T1DM and T2DM groups were also observed in the present study. Compared with individuals with T1DM (21.8%), a considerably higher percentage of patients with T2DM (63.6%) had obesity. This is in line with the pathophysiology of T2DM, which has a strong correlation with insulin resistance and obesity.^[25,26]^ Nonetheless, because T1DM is an autoimmune illness in which insulin shortage frequently results in weight reduction, patients were more likely to have a normal BMI or be underweight.^[27]^

Significant disparities were observed between the lipid profiles of patients with T1DM and T2DM, especially in terms of total cholesterol, LDL, HDL, and triglyceride levels. Compared with patients with T2DM, those with T1DM had lower mean triglyceride levels but higher mean LDL, HDL, and total cholesterol levels.

In particular, the mean HDL level was significantly higher in patients with T1DM (1.38 mmol/L) than in those with T2DM (1.14 mmol/L) (*P* < .001). However, the mean LDL levels were 3.03 and 2.91 mmol/L for patients with T1DM and T2DM, respectively (*P* = .03). Since LDL is referred to as “bad cholesterol” and raises the risk of atherosclerosis and cardiovascular illnesses, elevated LDL levels in T1DM are concerning.^[28]^ This discrepancy may be explained by the way that long-term diabetes affects lipid metabolism and by how insulin therapy works, as T1DM and T2DM may have differing effects on LDL levels.^[29]^ Given the elevated LDL levels in patients with T1DM, more aggressive lipid-lowering therapies may be required to reduce cardiovascular risk.^[30]^

HDL is often referred to as “good cholesterol” because of its heart disease-prevention properties and its role in the elimination of LDL from the bloodstream.^[31]^ Owing to variations in insulin sensitivity and treatment approaches, the increased HDL levels in patients with T1DM may indicate a more advantageous lipid profile in terms of cardiovascular protection.^[31]^ Despite the higher levels of LDL that have been reported, this contrast emphasizes the protective function of HDL in patients with T1DM.^[31]^

LDL, HDL, and other lipid components are included in total cholesterol, and higher total cholesterol levels in patients with T1DM indicate a higher overall blood cholesterol burden.^[32]^ This may be the result of longer-term diabetes and its effects on lipid metabolism, or T1DM and T2DM may respond differently to insulin therapy in terms of metabolism.^[32]^ To lower the risk of CVD, total cholesterol management is essential, particularly for patients with T1DM, who tend to have higher total cholesterol levels.^[32]^ Reduced HDL-C levels in patients with T2DM exacerbate the proatherogenic state. The altered metabolic state of T2DM, where insulin resistance is a prominent component that alters lipid metabolism, may be linked to this drop in HDL-C.^[33,34]^

Increased triglyceride levels in individuals with T2DM are known to be a risk factor for cardiovascular illnesses and are frequently linked to insulin resistance. Insulin resistance, which results in increased hepatic synthesis of VLDL and impaired lipolysis of circulating triglycerides, is frequently the cause of hypertriglyceridemia in individuals with T2DM. The presence of tiny, dense LDL particles (which are more atherogenic) is linked to elevated triglyceride levels. Moreover, residual particles (which are known to aid in the atherogenic process) can form as a result of elevated triglyceride levels.^[35,36]^ Patients with T1DM may have lower triglyceride levels due to improved metabolic control or distinct patterns of insulin sensitivity and lipid metabolism.^[36]^ This variation implies that patients with T2DM could need more targeted approaches to control triglyceride levels and lower cardiovascular risk, underscoring the significance of customized treatment plans for every form of diabetes.^[36]^

These findings are consistent with those of earlier research that patients with T2DM had a better lipid profile than those with T1DM, primarily when LDL is considered the major lipid target. These differences in lipid profiles are probably the result of insulin therapy and different metabolic profiles.^[37–39]^ The fact that most patients with T2DM use statins, whilst those with T1DM do not, could account for these results, as patients with T2DM have lower LDL levels than those with T1DM.^[40]^

While these lipid abnormalities are well-documented globally, the impact of ethnicity, genetic predisposition, and lifestyle factors on lipid metabolism in Saudi Arabian individuals must be considered. The Saudi population has distinct dietary habits, including a traditionally high intake of saturated fats and carbohydrates, which could influence lipid profiles differently compared to Western or East Asian populations. Furthermore, sedentary lifestyles and a high prevalence of metabolic syndrome in the region may exacerbate dyslipidemia, particularly in patients with T2DM. These factors may limit the direct applicability of our findings to other populations with different genetic backgrounds and lifestyle patterns. Future studies should explore ethnic and lifestyle-specific variations in lipid metabolism to develop more tailored therapeutic approaches.

The 2 groups also showed differences in the prevalence of lipid abnormalities. Compared with patients with T1DM, a significantly higher percentage of patients with T2DM had low HDL and aberrant triglyceride levels. Specifically, compared to 4.9% of patients with T1DM, 43% of patients with T2DM had aberrant triglycerides (*P* < .001), and 95.1% of patients with T2DM had low HDL levels (*P* < .001). This emphasizes the increased risk of CVD in patients with T2DM, which is worsened by dyslipidemia, characterized by high triglyceride and low HDL levels.^[15,41]^

Variations in lipid parameters across sexes were also observed. While patients with T1DM did not exhibit significant sex differences in these parameters, females with T2DM had significantly higher mean LDL and total cholesterol levels than males (*P* = .01). On the other hand, the mean HDL levels in females with T1DM and T2DM were higher than those in their male counterparts (*P* < .001). These results support the widespread observation that women, especially those with T2DM, are more likely to have higher levels of total and LDL cholesterol, possibly as a result of hormonal influences and variations in fat distribution.^[42–44]^

Significant clinical ramifications result from the variations in lipid profiles and the incidence of dyslipidemia between patients with T1DM and T2DM. Patients with T2DM have unfavorable lipid profiles, which increases their risk of cardiovascular complications. Therefore, rigorous cholesterol management (which includes medication and lifestyle changes) is essential. On the other hand, even if patients with T1DM are also at risk of CVD, they may benefit more from effectively managing their lipid levels by optimizing glycemic control and maintaining a healthy BMI.

Given the region-specific dietary and lifestyle habits in Saudi Arabia, healthcare providers should emphasize culturally appropriate lifestyle interventions, such as promoting traditional but healthier dietary modifications and increasing awareness of the importance of physical activity. Additionally, further research should focus on how genetic predispositions unique to the Saudi population affect lipid metabolism, helping refine personalized treatment strategies.

The advantages of this study include a large sample size and the utilization of comprehensive data from several primary healthcare facilities, which improve the generalizability of our conclusions. However, this study has some limitations. T1DM patients included in the study were adults 18 to 64 years, which omits the young population often affected by T1DM. The study’s cross-sectional design makes establishing a link between lipid abnormalities and diabetes type challenging. Furthermore, the reliance on telephone interviews and computerized medical records for data collection may have introduced recall bias and data inaccuracies. Further, longitudinal studies must validate these results and investigate the underlying mechanisms driving the observed lipid profile disparities between patients with T1DM and T2DM.

Moreover, another limitation is difference in the inclusion criteria between T1DM and T2DM participants which could impact lipid profiles and should be accounted for in statistical adjustments.

Another limitation is the lack of detailed stratification of participants based on ethnic background and lifestyle factors, which may influence lipid metabolism. While our findings are applicable to the Saudi population, they may not fully reflect the lipid profile variations seen in populations with different genetic backgrounds, dietary habits, or physical activity levels. Future research should incorporate multiethnic cohorts and assess the role of lifestyle behaviors in modulating lipid abnormalities in diabetes.

## 5. Conclusion

This study revealed notable distinctions in lipid profiles, BMI, glycemic control, and demographic traits between Saudi Arabian patients with T1DM and T2DM. Adverse lipid profiles and dyslipidemia were more prevalent in patients with T2DM, highlighting the need for focused therapies to reduce cardiovascular risk in this population. Understanding these distinctions is crucial in customizing therapeutic approaches and enhancing clinical outcomes for individuals with diabetes. This study provides valuable insights into the lipid profile differences between T1DM and T2DM patients in Saudi Arabia, highlighting the greater dyslipidemia risk in T2DM. However, limitations related to diagnostic criteria, and confounding factors should be addressed. Further investigation is necessary to investigate the long-term implications of these results and formulate practical management strategies for dyslipidemia in patients with diabetes.

**Table 4a:** Prevalence of lipid abnormalities, stratified by age group, in patients with T1DM.

Lipid type	Total n (%)	<40 years n (%)	≥40 years n (%)	*P*-value
LDL				
Normal	156 (30.6)	147 (30.6)	9 (32.1)	.86
Abnormal	353 (69.4)	334 (69.4)	19 (67.9)	
HDL				
Normal	467 (91.7)	440 (91.5)	27 (96.4)	.35
Abnormal	42 (8.3)	41 (8.5)	1 (3.6)	
Total chol				
Normal	392 (77.0)	373 (77.5)	19 (67.9)	.24
Abnormal	117 (23.0)	108 (22.5)	9 (32.1)	
Triglycerides				
Normal	484 (94.9)	457 (95.0)	27 (96.4)	.74
Abnormal	25 (4.9)	24 (5.0)	1 (3.6)	

HDL = high-density lipoprotein, LDL = low-density lipoprotein, T1DM = type 1 diabetes mellitus, T2DM = type 2 diabetes mellitus, total chol = total cholesterol.

**Table 4b:** Prevalence of lipid abnormalities, stratified by age group, in patients with T2DM.

Lipid type	Total n (%)	<40 years n (%)	≥40 years n (%)	*P*-value
LDL				
Normal	169 (41.3)	1 (8.3)	168 (42.3)	.02
Abnormal	240 (58.7)	11 (91.7)	229 (57.7)	
HDL				
Normal	20 (4.9)	1 (8.3)	19 (4.8)	.58
Abnormal	389 (95.1)	11 (91.7)	378 (95.2)	
Total chol				
Normal	310 (75.8)	2 (16.7)	308 (77.6)	<.001
Abnormal	99 (24.2)	10 (83.3)	89 (22.4)	
Triglycerides				
Normal	233 (57.0)	4 (33.3)	229 (57.7)	.09
Abnormal	176 (43.0)	8 (66.7)	168 (42.3)	

HDL = high-density lipoprotein, LDL = low-density lipoprotein, T1DM = type 1 diabetes mellitus, T2DM = type 2 diabetes mellitus, total chol = total cholesterol.

## Author contributions

**Conceptualization:** Moeber M. Mahzari.

**Data curation:** Ahmed R. Alibrahim.

**Formal analysis:** Ahmed R. Alibrahim.

**Investigation:** Weam S. Alharbi.

**Methodology:** Ahmed R. Alibrahim.

**Project administration:** Fatemah Saleh Bin Saleh.

**Resources:** Awad S. Alshahrani.

**Software:** Aida Aldughaither.

**Supervision:** Fatemah Saleh Bin Saleh.

**Validation:** Aida Aldughaither.

**Visualization:** Ghadah B. Alanazi.

**Writing – review & editing:** Fatemah Saleh Bin Saleh.

**Writing – original draft:** Ahmed R. Alibrahim.
